# Identification of a non-canonical G3BP-binding sequence in a Mayaro virus nsP3 hypervariable domain

**DOI:** 10.3389/fcimb.2022.958176

**Published:** 2022-08-11

**Authors:** Aymeric Neyret, Eric Bernard, Olivier Aïqui-Reboul-Paviet, William Bakhache, Patrick Eldin, Laurent Chaloin, Laurence Briant

**Affiliations:** Institut de Recherche en Infectiologie de Montpellier (IRIM), Université de Montpellier - CNRS, Montpellier, France

**Keywords:** Mayaro virus, alphavirus, G3BP, nsP3, host-pathogen interactions

## Abstract

Ras-GTPase-activating SH3 domain-binding-proteins 1 (G3BP1) and 2 (G3BP2) are multifunctional RNA-binding proteins involved in stress granule nucleation, previously identified as essential cofactors of Old World alphaviruses. They are recruited to viral replication complexes formed by the Chikungunya virus (CHIKV), Semliki Forest virus (SFV), and Sindbis virus (SINV) *via* an interaction with a duplicated FGxF motif conserved in the hypervariable domain (HVD) of virus-encoded nsP3. According to mutagenesis studies, this FGxF duplication is strictly required for G3BP binding and optimal viral growth. Contrasting with this scenario, nsP3 encoded by Mayaro virus (MAYV), an arthritogenic virus grouped with Old World alphaviruses, contains a single canonical FGxF sequence. In light of this unusual feature, we questioned MAYV nsP3/G3BPs relationships. We report that G3BP1 and G3BP2 are both required for MAYV growth in human cells and bind nsP3 protein. In infected cells, they are recruited to nsP3-containing cytosolic foci and active replication complexes. Unexpectedly, deletion of the single FGxF sequence in MAYV nsP3 did not abolish these phenotypes. Using mutagenesis and *in silico* modeling, we identify an upstream FGAP amino acid sequence as an additional MAYV nsP3/G3BP interaction motif required for optimal viral infectivity. These results, therefore, highlight a non-conventional G3BP binding sequence in MAYV nsP3.

## Introduction

Ras-GTPase-activating SH3 domain-binding proteins (G3BPs) are evolutionarily conserved multifunctional RNA-binding proteins, detected in humans, yeast, plants, and insects (in which they are referred to as Rasputin (Rin)) (Parker et al., 1996; Pazman et al., 2000; Krapp, 2017). In mammalian cells, these proteins are present in the form of two paralogs G3BP1 and G3BP2 which is separated into two spliced variants G3BP2a and G3BP2b (Kennedy et al., 2002). G3BPs are essential regulators of cellular RNA metabolism involved in transcript stabilization/decay (Martin et al., 2016; Fischer et al., 2020). They are also considered as critical nucleators of stress granules (SGs) where translation-stalled mRNAs complexed with RNA-binding proteins are accumulating in response to various cellular stresses (e.g., hypoxia, oxidative stress, and viral infections (Tourriere et al., 2003; Kedersha et al., 2016; Protter and Parker, 2016). In viral infections, assembly of these non-membranous cytosolic aggregates is part of the cellular responses mounted to block viral genome translation and subsequent progeny production. These compartments that sequester viral proteins and RNAs might also serve as platforms for the recruitment and activation of innate immune sensors (e.g., PKR, RIG-I, MDA5, cGAS) and antiviral proteins (e.g. TRIM5, ADAR1), therefore contributing to the initiation of an innate immune response (Onomoto et al., 2012; Law et al., 2019; Liu et al., 2019; Yang et al., 2019). To overcome such SG-associated antiviral responses and ensure productive infection, viruses have evolved a variety of mechanisms [reviewed in (Zhang et al., 2019)]. These strategies especially rely on the direct interaction and manipulation of G3BPs. This scenario has especially been reported for Old World (OW) alphaviruses, a group of mosquito-borne viruses in the *Togaviridae* family responsible for an acute musculoskeletal disease often evolving into chronic disease in humans. These pathogens, which include Sindbis (SINV), Semliki Forest (SFV), and Chikungunya (CHIKV) viruses, are positive-sense single-stranded RNA viruses. Their genome, characterized by a 5′-cap and a poly(A) tail, behaves as an mRNA. Upon delivery in the host cytoplasm, the alphavirus genome is translated into non-structural polyproteins targeted to the plasma membrane and sequentially processed into four non-structural proteins nsP1–4 that assemble themselves to produce the viral replication complex. Depending on the nsPs maturation state, this complex synthesizes a double-stranded RNA (dsRNA) replication intermediate and then uses this template to transcribe new positive-stranded viral genomes and a subgenomic RNA translated into structural proteins. NsP1, nsP2, nsP3, and nsP4 display methyl/guanylyltransferase, helicase/protease/NTPase, ADP ribosyl hydrolase, and RNA-dependent RNA polymerase activities, respectively (Kril et al., 2021). In addition, nsP3 supports the formation of a functional alphavirus replication machinery by recruiting a set of host-encoded factors depending on the host cell (Cristea et al., 2006; Gorchakov et al., 2008; Gotte et al., 2018; Meshram et al., 2018). This function was mapped in the intrinsically disordered C-terminal domain of nsP3 referred to as HVD for the “hypervariable domain,” which, despite being highly variable in size and showing very low identity even between closely related OW alphaviruses, mediates conserved interaction with host-encoded cofactors (Strauss et al., 1988; Gotte et al., 2018; Meshram et al., 2018). These include the four-and-a-half-LIM-only proteins FHL1, FHL2, and FHL3 (Meertens et al., 2019; Lukash et al., 2020), the SH3 domain-containing proteins CD2AP and SH3KBP1 (Mutso et al., 2018; Agback et al., 2019), the histone chaperones NAP1L1/4 (Meshram et al., 2018; Dominguez et al., 2021), the nucleolar transcriptional regulator MYBBP1A (Meshram et al., 2018), and the BIN-1/amphiphysin 2 F-BAR-containing protein (Neuvonen et al., 2011; Meshram et al., 2018). nsP3-HVD also contains a conserved duplicated FGxF motif that mediates binding to a hydrophobic pocket in the G3BP NTF2-like domain (Cristea et al., 2006; Panas et al., 2014; Panas et al., 2015; Schulte et al., 2016; Götte et al., 2019). As a consequence, CHIKV, SFV, and SINV nsP3 expressed in mammalian cells either as an ectopic protein or by an infectious virus recruit G3BPs to cytoplasmic aggregates, thereby preventing the G3BP-mediated nucleation of SGs (Fros et al., 2012; Panas et al., 2012). In infected cells, the FGxF tandem repeat also accounts for the redistribution of a fraction of G3BPs to cell membrane-anchored viral replication complexes where it is supposed to assist the assembly of an active viral replicase with negative-strand RNA synthesis activity (Gorchakov et al., 2008; Fros et al., 2012; Scholte et al., 2015; Remenyi et al., 2018; Götte et al., 2020), to protect the nascent genomic RNA (Kim et al., 2016), and to promote efficient translation of neosynthesized viral mRNAs by recruiting the translation initiation machinery (Götte et al., 2019). Mutational explorations revealed that duplication of the FGxF motif in nsP3-HVD is strictly required for G3BP binding, SG inhibition, and optimal OW alphavirus replication. Indeed, the deletion of even one of the two motifs, or phenylalanine-to-alanine substitution in these sequences, results in an attenuated SFV viral growth and non-viable CHIKV (Kim et al., 2016; Schulte et al., 2016; Meshram et al., 2018; Götte et al., 2019). The evolutionary conservation of this sequence repeat across Old World alphaviruses further supports its functional importance (Nowee et al., 2021). Despite how this sequence duplication impacts viral replication still remains elusive, structural studies recently revealed its requirement for assembly of higher-order oligomers of G3BP dimers interconnected by nsP3, probably required for efficient G3BP sequestration (Schulte et al., 2016).

Mayaro virus (MAYV) is an arthritogenic alphavirus endemic and enzootic in Pan-Amazonian countries, which is progressively extending to central/south America (Acosta-Ampudia et al., 2018) and is punctually imported in countries with a temperate climate (Hassing et al., 2010; Receveur et al., 2010). Phylogeny has grouped MAYV in the SFV serocomplex of alphaviruses (Lavergne et al., 2006). Its proximity with SFV and CHIKV was recently reasserted based on nsP3 amino acid sequence analysis (Nowee et al., 2021). However, contrasting with these viruses, the MAYV nsP3-HVD sequence only contains a single FGxF canonical motif, emphasizing the need to investigate MAYV requirement for G3BPs and the potential mechanisms contributing to G3BP/nsP3 interactions in this model. Here, we report that, while lacking FGxF duplication, MAYV depends on G3BPs for replication in mammalian cells. In infected cells, endogenous G3BP1 is recruited to nsP3-containing cytosolic foci and also frequently colocalizes with dsRNA replication intermediate as reported for other Old World alphaviruses (Gorchakov et al., 2008; Kim et al., 2016; Remenyi et al., 2018). We demonstrate that MAYV nsP3 indeed binds G3BPs. Unexpectedly, the mutation or deletion of the single FGxF canonical G3BP-binding sequence present in nsP3-HVD did not inhibit nsP3/G3BP interaction or colocalization, nor abolished MAYV infectivity when inserted in an infectious clone. Instead, the simultaneous deletion of an upstream FGAP sequence was required to abolish these. Altogether, these results show that MAYV nsP3 recruits G3BPs using an FGAP/FGDF tandem motif to promote viral replication in mammalian cells. Surprisingly, deletion of this sequence results in nsP3 nuclear accumulation. This study, therefore, sheds light on MAYV/host interactions required for replication in mammalian cells and brings novel information on the mechanisms evolved by OW alphaviruses to subvert SG components for their own genome replication.

## Results

### Identification of a vertebrate cell model for MAYV molecular studies

**Figure 1:**
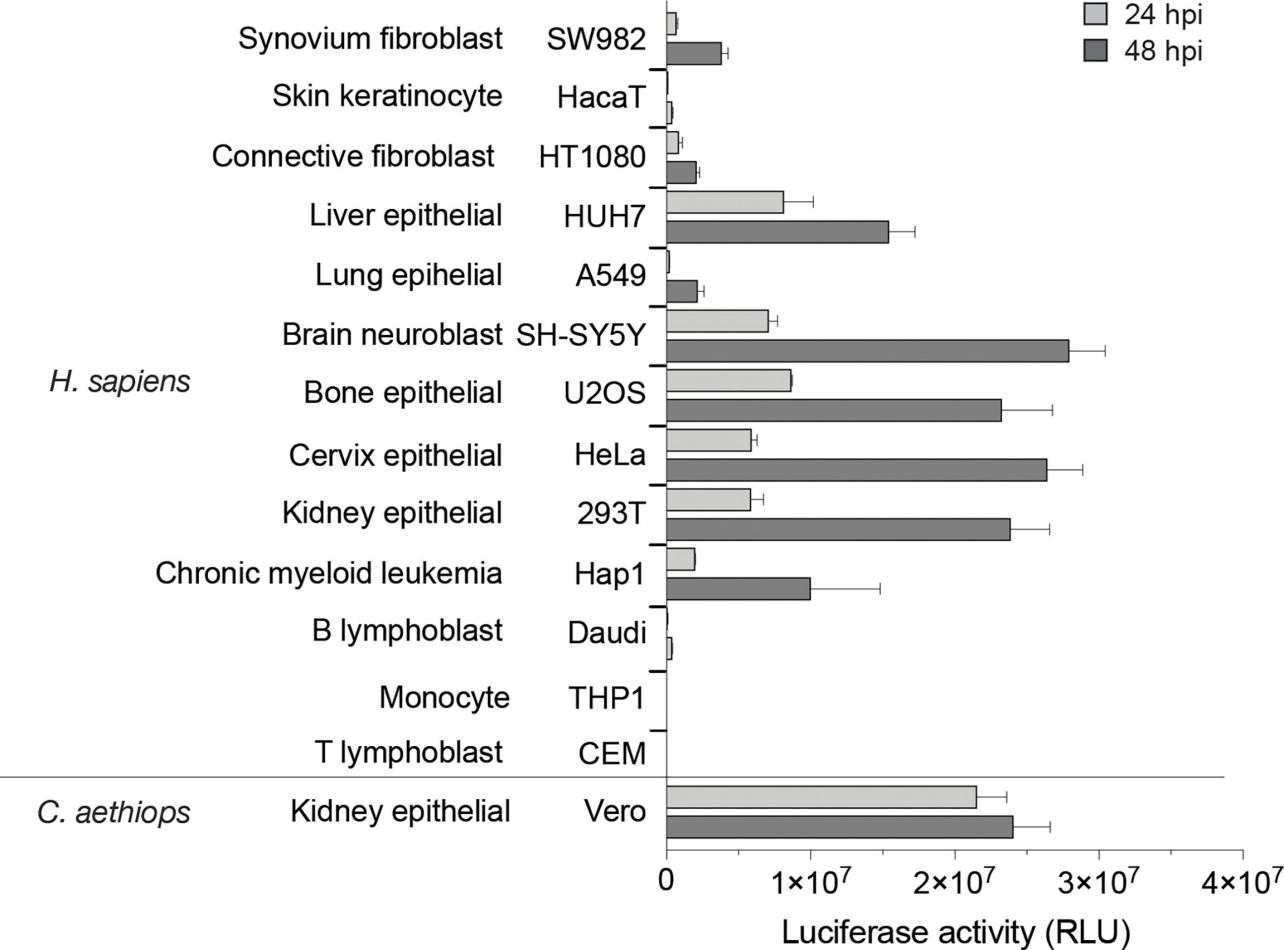
Susceptibility of vertebrate cell lines to MAYV infection. Cell lines were challenged with the MAYV-luc reporter virus used at an MOI of 0.1. Virus replication was monitored after 24 and 48 h by quantification of luciferase activity in cell lysates. Results are normalized according to protein concentration in the sample. Values are means of triplicates ± SD.

As MAYV is an understudied virus, we first sought to identify cell models useful for functional and molecular studies on this pathogen by testing its ability to replicate in a panel of immortalized vertebrate cell lines. For this, we took advantage of the previously reported MAYV-luc reporter virus developed from the TRVL4675 genotype D virus, bearing a *Renilla* luciferase reporter gene inserted within the C-terminal hypervariable domain of nsP3 (Chuong et al., 2019). Cells selected based on their relevance to MAYV-associated symptoms and according to their previous use in alphavirus research were challenged with the MAYV-luc reporter virus at an MOI of 0.1. Vero kidney epithelial cells derived from African green monkey, frequently used for alphavirus amplification, are shown as control. Expression of the virus-encoded luciferase gene was used as a simplified evaluation of virus spread in the cell culture. After 24 or 48 h of infection, most cell lines tested supported MAYV-luc replication (**Figure 1**). SH-SY5Y neuroblastoma cells and HEK293T and HeLa epithelial cells produced the highest luciferase levels. Cell lines relevant for alphavirus disease, namely, U2OS osteosarcoma cells, HUH7 hepatocytes, SW982 synoviocytes, and HT1080 fibroblasts, as well as the A549 pulmonary epithelial cells also supported detectable replication. Interestingly, the near-haploid HAP-1 iPSC-derived cell line was permissive for MAYV replication, therefore opening avenues for its utilization in genetic screens devoted to the study of MAYV/host interactions (Carette et al., 2011). Finally, HaCaT keratinocytes were poorly replicating MAYV thereby confirming the low susceptibility of this cell line to alphavirus replication (Bernard et al., 2014). As previously reported for CHIKV (Solignat et al., 2009), MAYV also poorly replicated in cells from the hematopoietic lineage, including T and B lymphoblasts and monocytes. According to these results, HeLa and HEK293T cells were selected as suitable cell models for functional studies of MAYV.

### G3BP1 and G3BP2 are critical for MAYV replication in vertebrate cells

**Figure 2:**
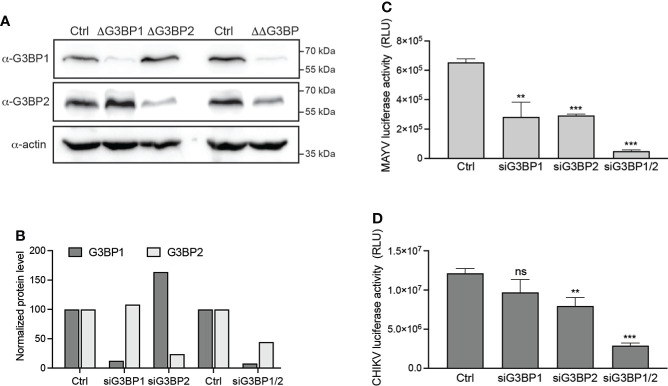
G3BP1 and G3BP2 are critical cofactors for MAYV replication. HeLa cells were transfected with nontargeting siRNA (Ctrl) or with siRNA targeting G3BP1 (siG3BP1), G3BP2 (siG3BP2), or both G3BP1 and G3BP2 (siG3BP1/2) as indicated. **(A)** After 48 h, cell lysates were separated by SDS-PAGE and probed for G3BP1, G3BP2, and actin. **(B)** Protein levels quantified from immunoblots using densitometry and normalized to actin levels were plotted. In parallel, the cells were infected with either **(C)** MAYV-luc or **(D)** CHIKV-nanoLuc viruses (MOI 0.1) for 24 h. Infection was monitored by quantification of luciferase activity in the cell lysates. Values were normalized according to protein content in the sample. Data are means of triplicates + SD and are representative of three independent experiments. ns, not significant.

We primarily investigated the G3BP requirement for MAYV replication using siRNA-mediated silencing followed by infection. HeLa cells were transfected with siRNA specific for G3BP1 (siG3BP1) or G3BP2 (siG3BP2) or a mixture of both siRNA (siG3BP1/2). After 48 h, immunoblot analysis assessed that GB3BP1 and G3BP2 expression was decreased by >80% by individual siRNA and the siG3BP1/2 cell displayed 92% G3BP1 and 56% G3BP2 protein level reduction (**Figures 2A, B**). siRNA-transfected cells were then infected with MAYV-luc at an MOI of 0.1. As a control, the same cells were infected with a CHIKV-nanoLuc virus using similar infection conditions. After 48 h, quantification of luciferase activity in the cell lysates revealed that MAYV replication was reduced by more than 50% in both siG3BP1 and siG3BP2 cells when compared with control cells. It was almost abolished (>97% reduction) in siG3BP1/2 cells (**Figure 2C**). CHIKV-nanoLuc replication was less dramatically reduced by individual siRNA but was decreased by >75% when G3BP1 and G3BP2 protein levels were simultaneously reduced as reported by others (Scholte et al., 2015; Kim et al., 2016) (**Figure 2D**). According to these results, MAYV replication in human cells requires both G3BP1 and G3BP2 expression and is dramatically reduced in cells depleted for both proteins, therefore paralleling CHIKV behavior and that of previously studied OW alphaviruses (Scholte et al., 2015; Kim et al., 2016).

### G3BP1 colocalizes with nsP3 and dsRNA in the cytoplasm of MAYV-infected cells

**Figure 3:**
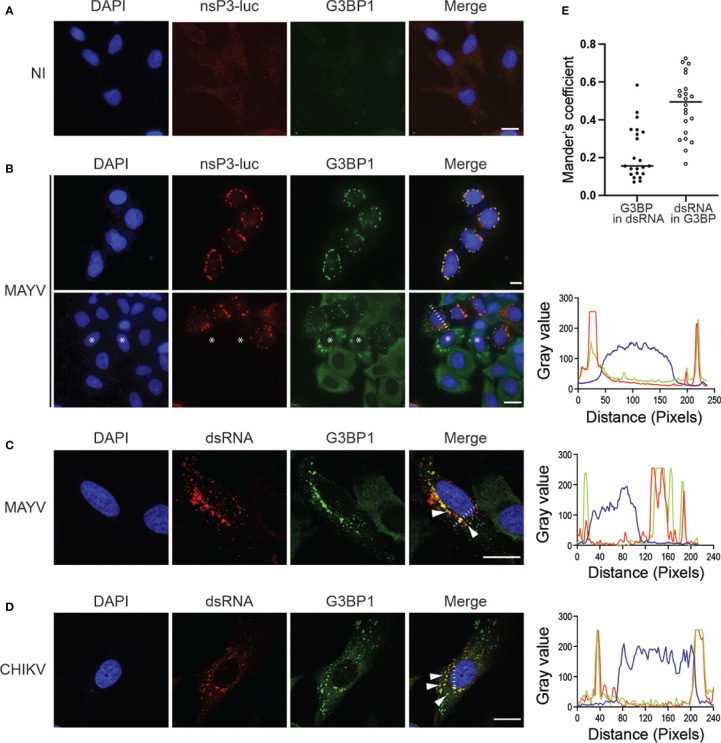
MAYV nsP3 and G3BP1 are colocalized to cytosolic granules in the cytoplasm of infected cells. HeLa cells were either left uninfected (NI) **(A)** or infected with MAYV-luc at a MOI of 0.5 for 24 h **(B)** and stained with antibodies for luciferase or G3BP1. Nuclei were strained with DAPI. Representative images are shown. * Indicates some uninfected cells. Cells infected with MAYV-luc **(C)** or CHIKV-nanoLuc **(D)** using the same experimental procedure were labeled for dsRNA, G3BP1, and nuclei were stained with DAPI. Foci of colocalized fluorescence are indicated by arrows. Scale bars = 20 µm. Fluorescence intensity profile corresponding to the white line is shown on the right. **(E)** Mander’s overlap coefficient was calculated between G3BP and dsRNA from experiment shown in **(C)** (n = 22 cells).

In alphavirus-infected cells, G3BPs are redistributed from a diffuse cytosolic pattern to discrete foci colocalizing with nsP3-containing cytosolic granules and with dsRNA-containing active replication complexes (Gorchakov et al., 2008; Kim et al., 2016; Remenyi et al., 2018). We therefore investigated G3BP subcellular localization in HeLa cells infected for 24 h with MAYV-luc. In the absence of commercially available anti-MAYV nsP3 antibodies, we used antibodies against luciferase to detect the virus-encoded nsP3-luciferase fusion protein. Confocal microscopy revealed that MAYV nsP3 expressed in infectious conditions forms large cytosolic foci, frequently located near the nuclear envelope (**Figure 3B**). In these cells, G3BP1 was re-localized from a diffuse staining observed in uninfected cells (**Figure 3A**) to cytosolic foci where it almost completely overlapped with nsP3 as attested by cross-sectional analysis of fluorescent signals (Mander’s overlap coefficient = 0.89). Unexpectedly, G3BP1 granular localization was sometimes detected in nsP3-negative cells. In this case, G3BP1 foci were larger and more irregularly shaped than those formed in nsP3-positive cells. This observation could reflect either a very early step of infection in which nsP3 is below our detection level or instead some paracrine response triggered by infection in uninfected bystander cells as recently reported (Iadevaia et al., 2022). Immunostaining of MAYV-infected cells with anti-dsRNA antibodies detected the viral replication complexes as punctate foci across the cytoplasm often colocalized with G3BP1 (Mander’s overlap coefficient 0.474 ± 0.16) (**Figure 3C**). By contrast, a significant fraction of G3BP1 did not overlap the dsRNA signal as previously reported by others (Mander’s overlap coefficient 0.222 ± 0.14) (Kim et al., 2016). This pattern was very similar to that observed for CHIKV-infected cells processed in the same conditions (**Figure 3D**). These observations therefore show that upon MAYV infection, G3BPs aggregate to nsP3-containing foci and to dsRNA-containing active replication complexes.

### MAYV nsP3 recruits G3BPs in the absence of a duplicated FGxF-binding motif

**Figure 4:**
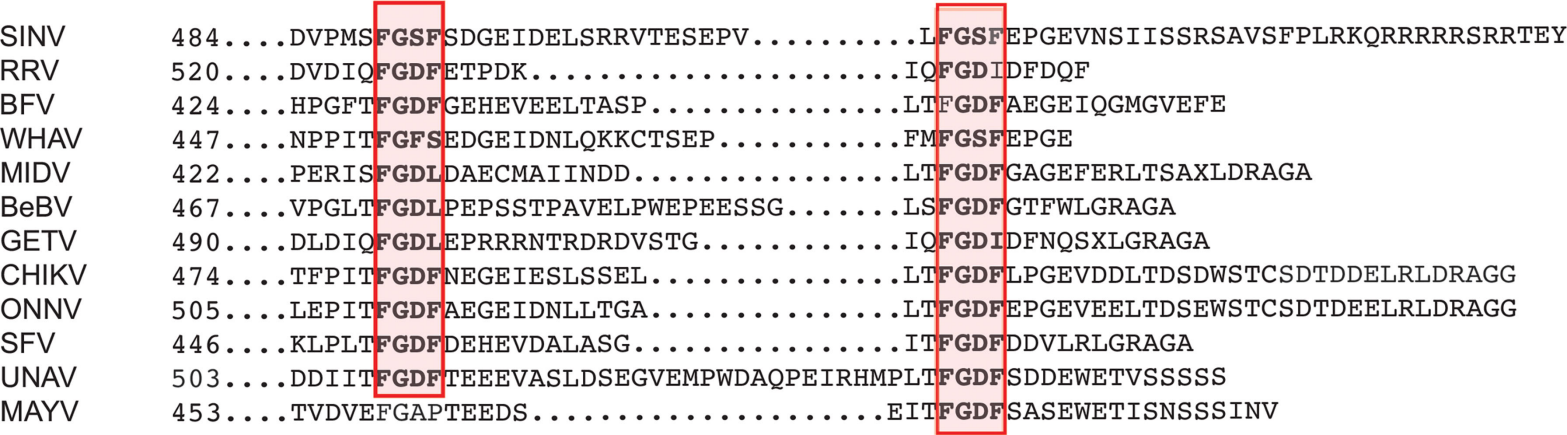
Conservation of G3BP canonical binding motifs in Old World alphavirus nsP3. The C-terminal sequence of nsP3 HVD encoded by alphavirus was aligned. The conserved canonical G3BP-binding sequences are boxed in red. SINV, Sindbis virus; RRV, Ross River virus; BFV, Barmah forest virus; WHAV, Whataora virus, MIDV, Middleburg virus; BeBV, Bebaru virus; GETV, Getah virus; CHIKV, Chikungunya virus; ONNV, O’nyong’nyong virus; SFV, Semliki Forest virus; UNAV, Una virus; MAYV, Mayaro virus.

**Figure 5:**
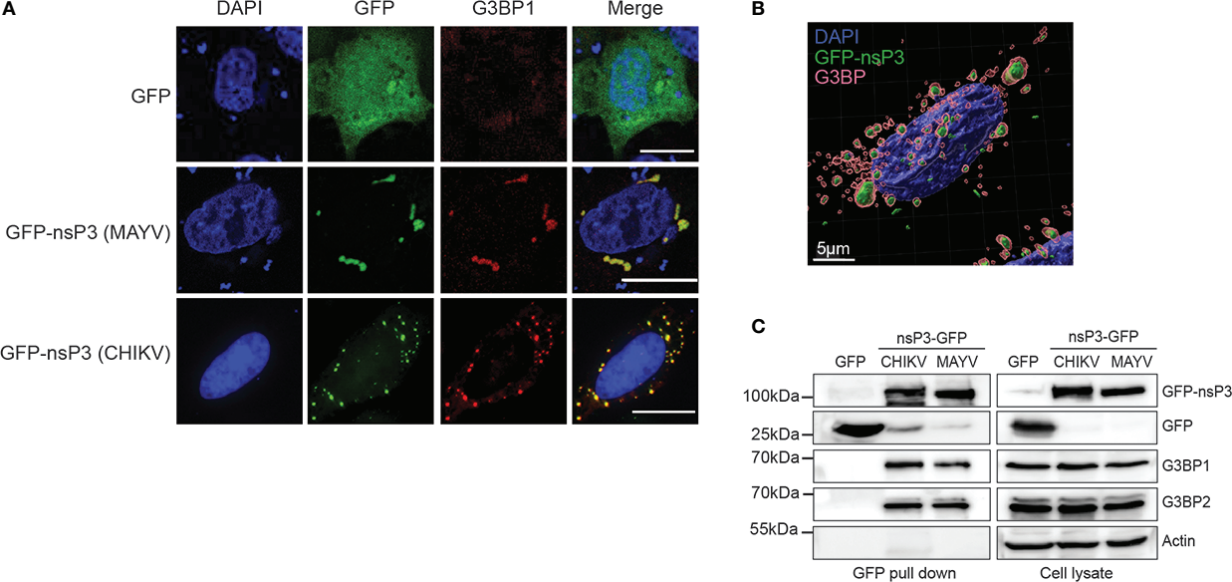
MAYV GFP-nsP3 interacts with G3BP and co-segregates in cytoplasmic foci. **(A)** HeLa cells transfected with GFP, MAYV GFP-nsP3, or CHIKV GFP-nsP3 expression vector were processed for immunofluorescence using anti-G3BP1 antibodies. Nuclei were visualized with DAPI staining, and cells were observed in confocal microscopy. Scale bars are 20 µm. **(B)** 3D image reconstruction was performed from confocal images and z-stacks acquired from cells expressing the MAYV GFP-nsP3 protein. **(C)** Total cell extracts prepared from **(A)** were pulled down using GFP-Trap. Lysates and pulled complexes were separated by SDS-PAGE and probed for GFP, G3BP1, G3BP2, and actin. Results are representative of two distinct experiments.

G3BP1 manipulation by SINV, CHIKV, and SFV alphavirus is mediated by the direct interaction of the G3BP NTF2-like domain with two conserved FGxF consensus motifs located in nsP3 HVD moiety (Panas et al., 2015; Schulte et al., 2016; Götte et al., 2019). In light of the reported critical requirement for this motif duplication, we interrogated its conservation in the MAYV nsP3 protein. The amino acid sequence of nsP3-HVD was aligned with that of other known OW alphaviruses replicating in mammalian cells (**Figure 4**). CHIKV and MAYV HVD sequences shared 24.7% amino acid identity (50.6% sequence similarity) compared with 64% identity and 88% similarity for nsP3 N-terminal moiety (not shown). We noticed that the FGxF repeat motifs, which constitute the G3BP-binding site of nsP3, are well conserved in the OW alphaviruses with limited variation at the fourth amino acid. Contrasting with all these viruses that contained two tandemly organized FGxF-like sequences, MAYV HVD contains a single FGDF motif. In light of this unusual feature, we questioned MAYV nsP3 direct contribution in G3BP relocalization. HeLa cells were transfected to express a GFP-fused nsP3 protein, stained with anti-G3BP1 antibodies, and processed for confocal microscopy. As observed in MAYV-infected cells (**Figure 3**), ectopically expressed GFP-nsP3 was detected as punctate and rod-like foci often located near the nucleus (**Figures 5A, B**). Moreover, endogenous G3BP1 was relocalized from a diffuse fluorescence observed in the control condition to cytosolic granular compartments almost completely colocalized with nsP3 (Mander’s overlap coefficient 0.923 ± 0.05). This phenotype was reminiscent of that detected in cells transfected to express CHIKV GFP-nsP3. To determine the ability of MAYV nsP3 to bind G3BPs, total cell extracts were prepared from the transfected cells and subjected to GFP pull-down. Immunoblotting of the precipitated complexes with G3BP1 or G3BP2 antisera revealed that both endogenous proteins co-precipitate with MAYV GFP-nsP3 as also observed for CHIKV GFP-nsP3 (**Figure 5C**). Altogether, these results support the fact that G3BP1 and G3BP2 both bind MAYV nsP3 and are recruited to cytosolic nsP3 aggregates in the absence of any other viral component.

### Identification of G3BP-binding elements in MAYV nsP3.

**Figure 6:**
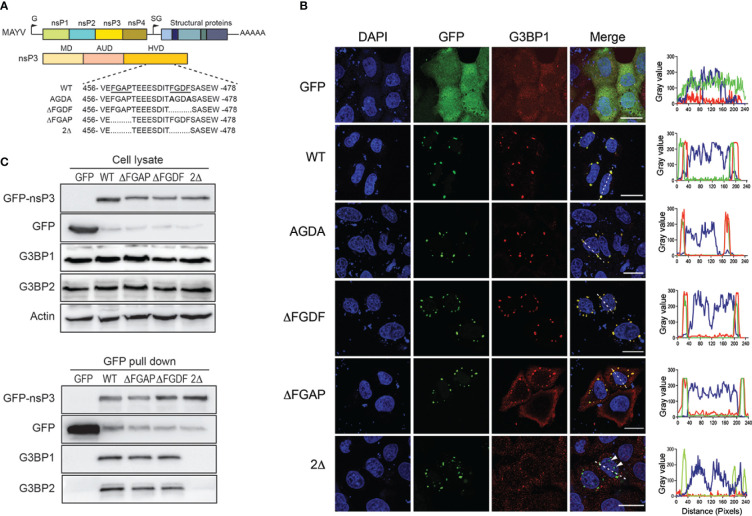
FGAP and FGDF sequences in MAYV nsP3 HVD are required for G3BP1 colocalization and binding. **(A)** Schematic representation of the MAYV genome, nsP3 organization, and deletion mutants in nsP3 HVD. The sequence of wild-type (WT) nsP3 with underlined FGAP and FGDF sequences is shown (top). Sequence and deleted amino acids in AGDA, ΔFGDF, ΔFGAP, and 2Δ mutants are represented. The numbers indicate the amino acid positions in the MAYV nsP3 sequence. **(B)** HeLa cells were transfected for 24 h with plasmids encoding GFP-nsP3 proteins depicted in **(A)**. The cells were labeled with anti-G3BP1 antibodies, and nuclei were stained with DAPI. An orthogonal fluorescence profile corresponding to the white line is shown for each panel. Bars, 20 µm. **(C)** Cell lysates prepared from **(B)** were pulled down using GFP-Trap. Total cell extracts (upper panel) and trapped complexes (lower panel) were separated on an SDS-PAGE and probed with antibodies against GFP, G3BP1, and G3BP2. Equivalent protein content in cell lysates was controlled using actin antiserum. Results are representative of two distinct experiments.

Alanine substitution of either phenylalanine in the OW alphavirus FGxF motif disrupts G3BP binding and nsP3/G3BP colocalization (Panas et al., 2015; Schulte et al., 2016). To decipher the role of the MAYV nsP3 single FGDF sequence in G3BP binding, we mutated this sequence into AGDA and tested the subcellular distribution of the mutant protein with respect to endogenous G3BP1 localization (**Figure 6A**). Unexpectedly, this mutant displayed a wild-type phenotype, forming cytoplasmic foci colocalized with endogenous G3BP1 (**Figure 6B**). This phenotype was also conserved for an nsP3 protein in which the FGDF sequence was deleted (ΔFGDF), suggesting that MAYV nsP3 binding to G3BPs either occurs independently of this FGDF sequence or requires the combined action of an additional binding motif. Previous mutagenesis studies of CHIKV nsP3 that confirmed the pivotal role of FGxF sequences also revealed the critical contribution of the 16-aa-long sequence upstream of these canonical G3BP-binding repeats (Meshram et al., 2018). Reanalyzing this sequence in MAYV sequence, we noticed the presence of phenylalanine and glycine forming part of a conserved FGAP sequence. Phenylalanine with glycine in the second position is critical for interaction with the NTF2-like sequence in G3BP (Schulte et al., 2016). To determine whether it may contribute to G3BP binding, the FGAP motif was deleted in the MAYV nsP3 sequence. In transfected cells, the corresponding ΔFGAP mutant formed cytosolic foci. Despite overlapping nsP3 staining in some instance, G3BP1 fluorescence was also detected as a diffuse signal in the cytoplasm, contrasting with profiles observed with the wild-type protein or other nsP3 mutants (**Figure 6B**). In light of this observation, we generated a 2Δ GFP-nsP3 protein lacking both the FGAP and FGDF sequences. This protein was detected as discrete cytosolic aggregates. In cells expressing the 2D GFP protein, endogenous G3BP1 was mostly detected as diffuse staining. Some G3BP1 cytosolic foci that did not overlap with nsP3 fluorescence were also observed in the cytoplasm, suggesting that the combined deletion of FGDF and FGAP sequences abolishes G3BP1 recruitment to nsP3 foci. To validate this hypothesis, GFP-nsP3 or mutant versions of this fusion protein were subjected to GFP pull-down and immunoblotted with anti-G3BPs antibodies. In these conditions, the ΔFGDF mutant coprecipitated with G3BP1 and G3BP2, attesting that the deletion of this single canonical G3BP binding sequence did not abolish interaction (**Figure 6C**). Similarly, ΔFGAP nsP3 interacted with G3BP1 and G3BP2. Contrasting with these results, the 2Δ mutant bearing a simultaneous deletion of the FGDF and FGAP sequences bound neither G3BP1 nor G3BP2. Altogether, these observations support the fact that MAYV nsP3 interacts with G3BP depending on the integrity of FGAP and FGDF sequences in its HVD. This result, therefore, identifies the FGAP sequence in MAYV HVD as a noncanonical G3BP-binding sequence acting in coordination with the single conventional FGDF sequence in this protein.

### *In silico* modeling of the MAYV nsP3:G3BP interaction

**Figure 7:**
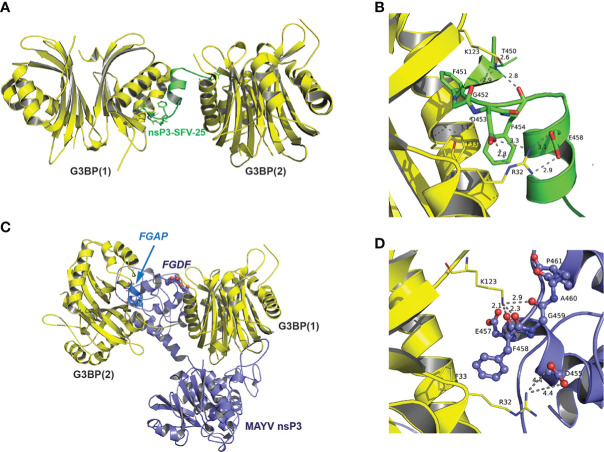
Predicted model of interaction between MAYV nsP3 and G3BP. **(A)** Crystal structure of G3BP dimers (depicted in yellow cartoon representation) in complex with nsP3-SFV-25 peptide (green) from PDB 5FW5 (second dimer added from the asymmetric unit). **(B)** Close-up view of atomic interactions between G3BP residues and nsP3-SFV-peptide within the FGDF motif. **(C)** 3D model of nsP3-MAYV (blue)/G3BPs (yellow) complex predicted by rigid docking. **(D)** Close-up view of interactions between G3BP and residues from FGAP motif of nsP3-MAYV (depicted in ball and stick).

The recent elucidation of the crystal structure of G3BP complexed with a 25-amino acid peptide derived from SFV nsP3-HVD has revealed the critical role of FGDF duplication in the assembly of G3BP aggregates (Schulte et al., 2016). According to this structure, each motif in this tandem repeat contacts the same hydrophobic pocket in the NTF2-like domain of two different G3BP molecules, thereby favoring the formation of a (G3BPs)2:nsP3 oligomer proposed as critical for the assembly of functional replication complexes at the plasma membrane. Based on our results highlighting the critical role of FGAP and FGDF motifs in G3BP interaction, we modeled this interaction *in silico*. The G3BP NTF2-like domain was considered a homodimer based on previous reports (Vognsen and Kristensen, 2012). As no experimental structure was available for full-length MAYV nsP3, a 3D model was built by homology with the SFV nsP3 N-terminal domain (residues 1–159) using the known NMR structure (Tsika et al., 2019), while the C-domain was predicted *ab initio*. Note that the lack of 3D information in this C-terminal moiety renders more delicate the prediction and should be taken with care. The rigid docking study was performed in two successive steps in order to form a tripartite complex between MAYV nsP3 protein and two G3BP dimers (**Figure 7**). First, the crystal structure of G3BP bound to the nsP3 SFV-25 peptide was used as a control and further analyzed to describe the main residues of G3BP interacting with the FGDF motif (**Figures 7A, B**). Two phenylalanine residues from SFV fit into a cavity formed by two short α-helices of G3BP including another Phe residue (Phe-33). Two strong interactions are also involved in this complex, one between Lys-123 and backbone oxygens of Gly-452 and Phe-454 and one salt bridge between Arg-32 and Asp-453/Glu-458 (**Figure 7B**). From rigid docking studies with nsP3-MAYV, the main population of predicted complexes was reproducing the interaction with the canonical FGDF motif, and an additional G3BP docking predicted the interaction with the noncanonical FGAP motif (**Figure 7C**). A closer view indicates that key residues of G3BP (Arg-32 and Lys-123) formed salt bridges and hydrogen bonds with the FGAP motif, with additional hydrophobic contacts mediated between Phe-458 from this motif and Phe-33 from G3BP similarly to that observed in the crystal structure (**Figure 7D**). Note that the quite long distance (4.4 Å) for Arg-32-Asp-455 interaction was labeled on purpose as it could be stronger if side-chain flexibility could have been introduced in the rigid docking. Interestingly, the Glu-457 residue located near Lys-131 and just preceding the FGAP motif could also contribute to strengthening the complex. Therefore, this *in silico* simulation further corroborates the capacity of FGAP and FGDF sequences in MAYV nsP3-HVD to mediate G3BPs dimer binding.

### Loss of G3BP binding relocalizes MAYV nsP3 to the nuclear compartment

**Figure 8:**
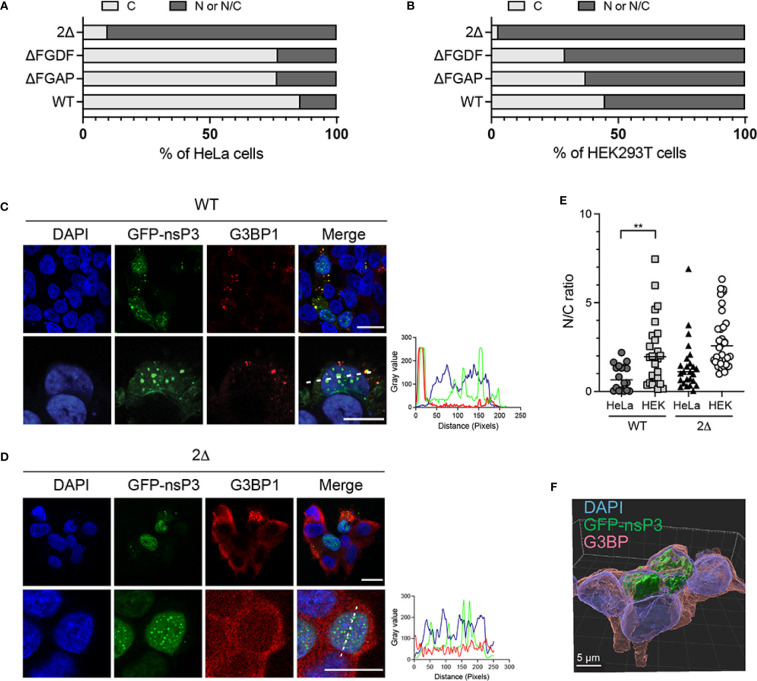
Subcellular distribution of WT and mutant GFP-nsP3 is determined by G3BP binding. HeLa cells **(A)** or HEK293T cells **(B)** transfected to express GFP-fused WT nsP3, or ΔFGAP, ΔFGDF, and 2Δ derivatives were examined by confocal microscopy. Fluorescence distribution was classified as exclusively cytoplasmic **(C)**, or present at least in the nucleus (N/C). Percentages of cells within each group were plotted (total number of cells _50 for each condition). Representative confocal images of HEK293T cells expressing the wild-type nsP3 **(C)** or the 2Δ mutant **(D)** are shown. Scale bars are 20 µm. **(E)** Nucleus-to-cytoplasm-fluorescence ratios were calculated from individual cells. **(F)** Z-stack analysis and 3D image reconstruction of 2Δ GFP-nsP3 and G3BP fluorescence in HEK293T cells.

Having a closer examination of the 2Δ nsP3 mutant outcome upon ectopic expression, we noticed that in a significant number of cells, this protein aggregated into nuclear foci (**Figure 6B**). We, therefore, reinvestigated the behavior of GFP-nsP3 and its mutants to provide a statistical view of their subcellular distribution. From an analysis of >50 HeLa cells, we observed that GFP-nsP3 was generally detected in the form of cytoplasmic foci (**Figure 8A**). Rarely, this fusion protein was detected in the nucleus of transfected cells, either in the form of a diffuse fluorescence or forming punctate aggregates. The ΔFGDF and ΔFGAP nsP3 mutants were also mostly localized to the cytoplasm in a similar ratio as the wild-type protein, indicating that the individual deletion of these sequences did not change nsP3 subcellular localization. Contrasting with this result, the 2Δ nsP3 was rarely exclusively cytoplasmic. Instead, in >90% of the cells examined, this mutant was detected at least in part in the nucleus. To investigate whether these phenotypes depended on a particular cell type, we repeated these experiments in HEK293T cells. In this cell line, the wild-type nsP3 was more frequently detected in the nucleus than observed in HeLa cells (**Figures 8B, C**). Indeed, the fluorescence was exclusively cytosolic in only _˜_45% of the cells observed. In 20% of the cells, GFP-nsP3 was exclusively observed in the nucleus. This tendency was confirmed calculating the nuclear-to-cytoplasmic-fluorescence ratio (**Figure 8E**). In these conditions, the ΔFGDF and ΔFGAP mutants behaved similarly to the parental protein. Finally, the 2Δ nsP3 was very frequently (_˜_95%) detected in the nucleus of transfected cells (**Figure 8D, E**), as confirmed by z-stack analysis and 3D image reconstruction (**Figure 8F**). Altogether, our experiments indicate that nsP3 subcellular localization may differ according to the cell model considered and is also altered by deletion of both G3BP-binding sites.

### Functional importance of the FGAP motif in MAYV infection

**Figure 9:**
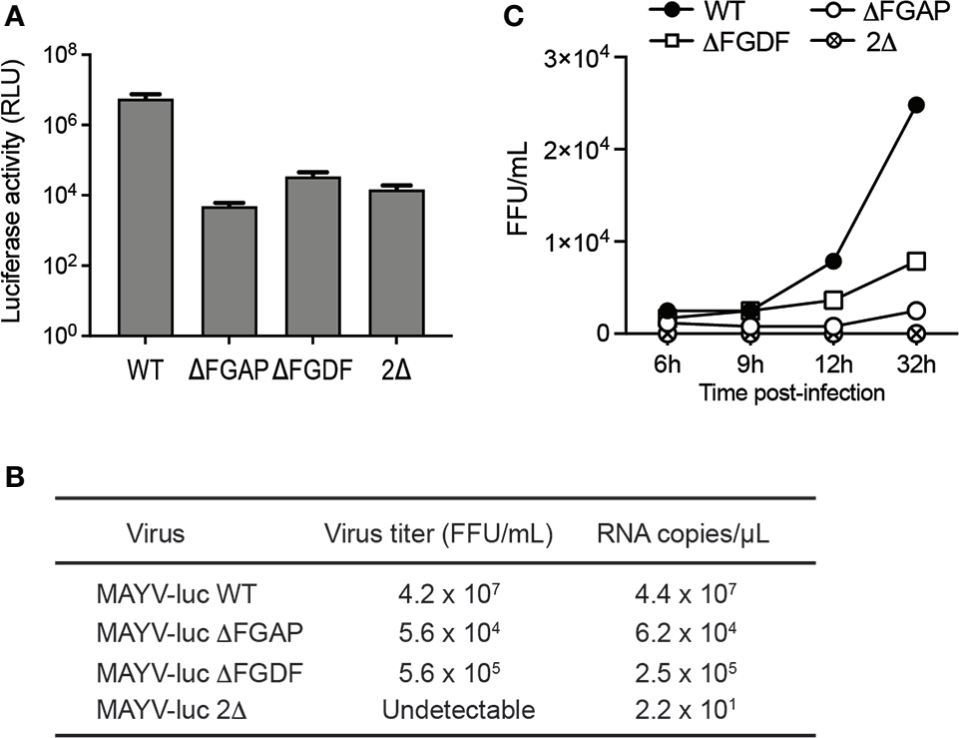
Contribution of FGAP and FGDF motifs to MAYV replication. **(A)** HEK293T cells were transfected with WT, ΔFGAP, ΔFDF, or 2Δ MAYV infectious clones. At 24 h post-transfection, plasmid expression was controlled by determination of luciferase activity in the cell lysates. Data are averages of two experiments, and error bars are standard deviations. **(B)** Genome equivalent in supernatants was quantified by RT-qPCR, and infectious titers were determined in parallel. **(C)** Multiple step growth curves were determined on HeLa cells infected at a MOI of 0.01. At 6, 9, 24, and 32 h postinfection, supernatants were collected, and viral titers were determined.

Previous investigations reported the critical role of the FGDF tandem repeat in alphavirus infectivity. Deletion of any G3BP-binding sequence resulted in an attenuated SFV replication in vertebrate cells and in nonviable CHIKV (Panas et al., 2015; Schulte et al., 2016). To determine the biological significance of the nonconventional G3BP-binding sequence identified in MAYV nsP3-HVD, we generated MAYV-luc mutant viruses carrying a deletion of FGDF (ΔFGDF), FGAP (ΔFGAP), or both motifs (2Δ). After transfection of HEK293T cells, mutant viral genomes were efficiently transcribed and translated according to genome-directed luciferase reporter gene expression in the cell lysate, albeit reduced by over 2Log compared with the parental virus (**Figure 9A**). Infectious titers of the corresponding supernatants were determined against Vero cells, and the RNA genome content in the inoculum was quantified by RT-qPCR (**Figure 9B**). In these conditions, the ΔFGDF and ΔFGAP mutants showed a two-to-three-order-of-magnitude reduction in viral titers and genome copy numbers compared to wild-type MAYV, while the 2Δ mutant was nonviable. Multiple-step growth curve experiments were performed in order to determine whether nsP3 deletions affect viral infectivity (**Figure 9C**). HeLa cells were infected with a normalized amount of wild-type, ΔFGAP, or ΔFGDF-mutant viruses diluted to an MOI of 0.01, or with undiluted culture supernatant harvested from the 2Δ condition. Viral titer determination in culture supernatants at 6, 9, 24, and 32 h after infection revealed that ΔFGAP and ΔFGDF MAYV replicated more slowly than the parental virus. In these conditions, the ΔFGDF mutant showed a slight replicative advantage when compared with the ΔFGAP MAYV. Again, no infectious particle was recovered from the 2Δ-mutant condition. Altogether, these results indicate that the integrity of both canonical FGDF and noncanonical FGAP binding sequences is required for optimal infection. They also argue for a possible predominant requirement for the FGAP motif over the FGDF sequence for viral replication.

## Discussion

The intense research effort devoted to the study of alphavirus interaction with their host has identified G3BPs as essential cell-encoded cofactors recruited to viral replication complexes (Cristea et al., 2010; Fros et al., 2012; Panas et al., 2012; Scholte et al., 2015; Kim et al., 2016; Schulte et al., 2016; Götte et al., 2019). Molecular investigations performed on a few model viruses (CHIKV, SINV, and SFV) have established the strict requirement for a conserved duplicated FGxF motif in nsP3-HVD to divert G3BPs from their SG assembly function and to assemble a functional replicase. According to structural modeling, the essential need for this FGxF tandem repeat is supposed to reflect its contribution in the assembly of (G3BPs)2:nsP3 polycomplexes in which each FGxF motif binds a separate G3BP dimer (Götte et al., 2019). The infectivity loss observed upon deletion of one of the two FGxF motifs (Panas et al., 2015) and sequence conservation of evolution across OW alphaviruses further support the essential functional need for FGxF motif duplication. This motif duplication, with limited variation at the fourth amino acid position (FGx[I/L/F]), is indeed detected in nsP3-HVD from almost all alphaviruses replicating in humans. Contrasting with this knowledge, MAYV, despite being grouped in the SFV serocomplex, contains a single FGxF motif, raising the question of its G3BP dependency for replication and of the eventual molecular mechanisms accounting for nsP3/G3BP binding. Here, we report that MAYV has evolved an FGAP sequence in nsP3 HVD to assist its single FGDF motif for G3BPs binding. Both motifs are required to ensure optimal MAYV infectivity.

The presence of a single FGxF sequence is a peculiarity of MAYV nsP3, which is conserved in the sequence of all isolates sequenced so far, including the ancestral TRVL4675 strain, used in this study, and isolates more recently detected in various countries across South America. This conservation is also observed whether these strains were grouped with genotype D like TRVL4675 or with genotype L, which are >15% divergent at the nucleic acid level (Chandler et al., 2006). Compared with other known alphaviruses, a single FGxF motif was only detected in the sequence of mosquito-restricted alphaviruses, namely, the Mwinilunga virus (MWNV) and the Taï Forest (TALV) virus recently reported unable to recruit G3BPs in cytosolic foci (Nowee et al., 2021). MAYV is therefore the only known human-adapted OW alphavirus with such characteristics. Despite this singularity, we observed that MAYV nsP3 binds G3BP1 and G3BP2 paralogs that are recruited to replication complexes in infected cells and are strictly required for MAYV infectivity. Based on this evidence, we undertook a mutagenesis approach to validate the role of the single MAYV FGDF in G3BP binding. Contrary to other studied OW alphaviruses, phenylalanine mutation or total deletion of this single motif in MAYV did not abolish nsP3/G3BP colocalization in cytosolic granules or co-immunoprecipitation of these proteins (Schulte et al., 2016), suggesting that G3BP interaction with MAYV HVD relies on additional noncanonical interaction sequence(s). Our search for such motif(s) was guided by the examination of sequence conservation in known cellular and viral G3BPs-binding partners. The cell nucleoporin Nup62 (FxFGxF) and nsP3 encoded by CHIKV and SFV (FGDF), SINV (FGSF), GETV (FGDI/L), and RRV (FGDF/I), as well as nonstructural proteins from plant viruses in the *Nanoviridae* family (FGEF), all display strict conservation of a phenylalanine/glycine di-amino-acid sequence combined with an acidic environment (Clarkson et al., 1996). This sequence, together with the phenylalanine side chains, plays a critical role in contacting G3BPs as attested by mutagenesis studies and crystal structure analysis of NTF2-like domains with its ligands (Kristensen, 2015; Schulte et al., 2016). According to this information, we explored the functional importance of a conserved FGAP sequence located upstream of the FGDF consensus motif in MAYV nsP3-HVD. The slightly reduced G3BP-binding capacity observed upon deletion of the FGAP sequence encouraged us to combine this deletion with that of the FGDF motif, which did not by itself prevent G3BP interaction. MAYV nsP3 simultaneously lacking FGAP and FGDF sequences was unable to recruit and bind G3BPs, attesting to the combined role of these motifs. This result therefore identifies the FGAP motif as a previously unreported G3BP binding sequence that differs from the canonical FGxF tandem repeat in which the phenylalanine conservation at position 4 is critical for OW alphavirus infectivity (Panas et al., 2015). The existence of the nonconventional G3BP-binding motif was previously highlighted by the study of the Eastern equine encephalitis virus (EEEV) and of the Western equine encephalitis virus (WEEV), both grouped as encephalitic New World alphaviruses, interacting with G3BPs despite the absence of any FGxF sequence in nsP3-HVD (Kim et al., 2016; Frolov et al., 2017; Nowee et al., 2021). Such feature could also be under-evaluated in OW alphaviruses. Indeed, Meshram et al. (Meshram et al., 2018; Dominguez et al., 2021) recently reported that the 16-amino-acid domain upstream the well-defined FGDF tandem repeat in CHIKV nsP3, which contains an FGAP sequence, behaves as an additional G3BP-binding sequence (Meshram et al., 2018; Dominguez et al., 2021). Contrasting with MAYV, its contribution in G3BPs binding might, however, be accessory in this model due to the presence of the FGxF tandem sequence.

Our *in silico* simulations corroborate the potential contribution of the MAYV FGAP sequence in binding the G3BP NTF2-like domain. According to these predictions, the missing FGDF motif is similarly replaced by the FGAP motif, and the analysis of interactions formed between G3BP and nsP3 residues showed the implications of the same G3BP key residues (Lys-123 and Arg-32). However, comparing the SFV FGxF and MAYV FGAP interaction with G3BP revealed a reverse orientation. Moreover, the unique “F” present in the MAYV sequence was found to behave as the “F” residue at position 4 in the SFV FGxF motif. Interestingly, the Glu-457 residue upstream this motif was found to compensate for the absence of the Asp residue from the FGDF consensus motif, contributing in interactions with the basic residues in the N-terminus of G3BPs (Panas et al., 2015). Altogether, our predictions strongly suggest that the FG sequence in the full motif is essential for G3BP binding and represents the most important recognition signal with the assistance of the surrounding negatively charged amino acids. Based on these differences, the FGAP sequence in MAYV therefore likely represents an evolutionary distinct G3BP-binding motif taking benefit of a more flexible motif (including FG and other negatively charged residues), allowing to fulfill the biological function of the canonical FGDF motif. This motif may therefore assist the single FGDF motif for G3BP interaction and assembly of high-order G3BP/nsP3 polycomplexes that prevent SG Formation and contribute to the assembly and function of Old World alphavirus replication complexes (Scholte et al., 2015; Kim et al., 2016; Remenyi et al., 2018; Götte et al., 2020). Interestingly, deletion of the FGAP motif impacted more MAYV infectivity than observed for the FGDF deletion. This result could be reminiscent of the previously reported hierarchical binding mode of the duplicated FGxF motifs to G3BPs in which the N-terminal prevails over the C-terminal FGxF-binding sequence for viral infectivity (Schulte et al., 2016).

Finally, an unexpected observation from this study refers to unusual subcellular localization of the 2Δ nsP3 mutant that was frequently detected in the nucleus of transfected cells. OW alphavirus nsP3 are well described as cytoplasmic proteins. Here, we detected the wild-type MAYV nsP3 as dispersed cytosolic aggregates sometimes showing a perinuclear localization. By contrast, deletion of the FGAP and FGDF motifs, resulting in abolition of G3BP binding, frequently redirected nsP3 to the nucleus. Recently, Nowee et al. (2021) reported that nsP3s encoded by BEBV, GETV, BFV, SINV, WHAV, and SESV, despite cytoplasmic in wild-type cells, are relocalized to the nucleus upon G3BPs KO. Our study of the 2Δ nsP3 mutant extends this observation to MAYV and suggests that in this model, G3BP-binding capacity could be critical for nsP3 cytosolic targeting. Interestingly, G3BPs naturally function as a transport factor for RNA between the cytoplasm and the nucleus and bind the nucleoporin p62 (Fribourg et al., 2001). The exact role of G3BPs in nsP3 appropriate localization, therefore, deserves attention.

## Material and methods

### Cells

SW982 cells were purchased from the CLS Cell Lines Service GmbH cell repository service. HaCaT cells were kindly provided by Pr. Laurent Meunier (Cellular Pharmacology, Montpellier). Hap1 cells are a gift of Thijn Brumelkamp (Oncode Institute, Netherlands). Other cell lines were obtained from ATCC (VA 20110, USA). Adherent cells were cultured in Dulbecco’s modified Eagle’s medium (DMEM, Thermo Fischer Scientific) supplemented with 1% penicillin/streptomycin and 10% fetal calf serum (FCS, Lonza) and grown at 37°C in a 5% CO_2_ atmosphere. Lymphocytes and macrophages were cultured in RPMI in place of DMEM.

### Viruses and infections

The MAYV-luc derived from the TRVL 4675 strain and CHIKV-nanoLuc reporter viruses were previously reported (Matkovic et al., 2018; Chuong et al., 2019). Viral stocks were produced by transfection of the infectious clones in HEK293T cells using jetPEI (Polyplus Transfection). Forty-eight hours after transfection, the supernatant was collected, filtered through a 0.45-µm membrane, and titered using a TCID_50_ assay. For infections, the cells (70%–80% confluence) were infected with MAYV-luc diluted to achieve the desired MOI. After 24 h in culture, the cells were lysed with Passive Lysis Buffer (Promega). Replication was monitored using the Genofax C luciferase assay kit (Yelen Analytics). Luminescence was measured directly from the cell lysate using a Spark 10M fluorometer (Tecan). Values were normalized according to protein content in the sample determined using the BCA Assay (Pierce).

### Growth curves

Cells grown in 96-well dishes to ~90% confluence were infected at an MOI of 0.01. Supernatants were collected at 6, 9, 24, and 32 h postinfection. Virus titers were determined on Vero cells using a TCID_50_ assay determined from the Spearman–Karber method. Values were converted in FFU/mL assuming that 1 PFU/ml = 0.56 TCID_50_.

### RT-qPCR quantification of viral genomes

For RT-qPCR, viral supernatants were ultracentrifuged at 95,000 rpm for 10 min using a TLA 100.2 rotor (Beckman Coulter) and lysed with the Luna^®^ Cell Ready lysis module (New England Biolabs). The amplification reaction was run on a LightCycler^®^ 480 thermocycler (Roche Diagnostics) using the Luna^®^ Universal One-Step RT-qPCR Kit (New England Biolabs), and MAYV_For: 5′-TTGTGGCCGAGAGTTCAGG and MAYV_Rev: 5′-AGCATCACTTTTTCGCACC primers. Serial dilutions of the MAYV-luc plasmid were used for the standard range. Each qPCR was performed in triplicate, and the means and standard deviations were calculated.

### Plasmids

The nsP3 sequence was amplified by PCR from the MAYV-luc infectious clone (Chuong et al., 2019) using the primers Fw: 5′-ctcgagccgctcccgcttacactgtcaa and Rev: 5′-gggccctcaggatgagttactgatggtctcc and cloned into the pEGFP-C1 plasmid. Mutants were generated using the QuickChange mutagenesis kit (Agilent) and the following primers: AGDA 5′-atagtgaaatcacagcaggagacgcatctgcttcggagtg and 5′-cactccgaagcagatgcgtctcctgctgtgatttcactat; ΔFGAP 5′-gaccgtcgacgtagagaccgaagaggatagt and 5′-actatcctcttcggtctctacgtcgacggtc; ΔFGDF 5′-ggatagtgaaatcacatctgcttcggagtggg and 5′-cccactccgaagcagatgtgatttcactatcc, or a combination of primers. Plasmids were transfected using jetPEI reagent (Polyplus Transfection). Infectious clones were constructed as follows. A PCR fragment amplified from the MAYV-luc infectious clone using the following primers 5′-tgctaagaattcgcatcaccggcacagagtac and 5′-tgctaagaattcgcatcaccggcacagagtac was introduced into the pcDNA3.1 plasmid and mutated as described above. The *EcoNI*-*BlpI* fragment was subcloned into the parental infectious clone. Plasmids were sequenced to confirm the presence of mutations.

### siRNA and transfections

siRNA targeting G3BP1 or G3BP2 were purchased from Sigma-Aldrich. Nontargeting control siRNA were from Dharmacon. Transfection of 75 nM siRNA was achieved using INTERFERin (Polyplus Transfection). At 48 h post-transfection, an aliquot of the cells was harvested to determine the silencing efficiency by immunoblotting. The remaining cells were plated and infected with MAYV-luc.

### Immunofluorescence microscopy and image analysis

Cells grown on glass coverslips were washed with PBS and then fixed with 4% formalin (Sigma Aldrich) for 1 h at room temperature. For intracellular labeling, the cells were permeabilized with 0.1% Triton X-100 in PBS and blocked with 0.2% bovine serum albumin and 0.05% Tween 20 in PBS before incubation with primary antibodies for 1 h at room temperature. Antibodies used were anti-G3BP1 (#SC365338, Santa Cruz Biotechnologies), anti-G3BP2 (#16276-1-AP, Proteintech), and anti-luciferase (MAB10026, R&D Systems). The J2 monoclonal antibody (Scicons) was used to detect dsRNA. Secondary reagents (anti-mouse and anti-rabbit antibodies conjugated with Alexa488, Alexa594, or Alexa647) were added for 1 h at room temperature, and nuclei were stained with DAPI (Sigma-Aldrich). After final washes, coverslips were mounted with ProLong Gold Antifade mounting media (Thermo Fisher Scientific). Images were acquired by confocal laser scanning microscopy using a Leica SP5-SMD scanning confocal microscope equipped with a 63×, 1.4 numerical aperture Leica Apochromat oil lens at the Montpellier Resources Imaging platform. Image analysis was performed utilizing the ImageJ software and the JACoP plugin. 3D reconstruction was performed by the Imaris software. The ratio of nuclear to cytoplasmic fluorescence was calculated for individual cells using ImageJ. All images are individual cross-sections except when specified in the text.

### Immunoblotting and immunoprecipitation

Cells were lysed on ice in 50 mM Tris–HCl (pH 8.0), 100  mM NaCl, 1  mM MgCl_2_, 1% Triton X-100, and protease inhibitors (Complete; Roche) and clarified by centrifugation at 10,000 × g for 30 min. Fifty micrograms of total protein was kept as an input, and 400 µg cell lysate was incubated with GFP-Trap Magnetic Agarose (ChromoTek) for 2 h at 4°C. The bead-immune complexes were separated, washed three times with lysis buffer, and resuspended in Laemmli buffer. Immunoprecipitates and whole-cell lysates were separated by SDS-PAGE and then transferred to a polyvinylidene fluoride membrane (Hybond, Amersham). Membranes were blocked against nonspecific binding by using 0.5% casein and 0.1% Tween 20 in PBS and probed with appropriate primary antibodies against G3BP-1 (#SC365338, Santa Cruz Biotechnologies), G3BP-2 (#16276-1-AP, Proteintech), anti-GFP (Santa Cruz Biotechnologies), anti-actin (A3854, Sigma-Aldrich), and nsP3 rabbit polyclonal serum (kindly provided by Andres Merits, University of Tartu, Estonia). After extensive washings, the membranes were probed with HRP-conjugated secondary antibodies. Chemiluminescence was detected with Luminata Forte (Merck) using a ChemiDoc (Bio-Rad). Densitometry was performed using the Fiji software (ImageJ).

### Sequence alignment

The following are the viruses’ corresponding GenBank accession numbers: Sindbis virus (NC_001547.1); Ross River (NC_001544.1); Barmah Forest virus (NC_001786.1); Whataroa virus (NC_016961.1), Middelburg virus ZRU080/14 isolate (MT015693.1), Bebaru virus (NC_016962.1), Getah virus GERV-V1 isolate (KY399029.1), CHIKV LR-OPY1 strain (EU224268.1), O’nyong’nyong virus, (NC_001512.1), Semliki Forest virus (NC_003215.1), Una virus (NC_043403), and Mayaro virus TRVL4675 strain (MK070492.1).

### *In silico* modeling of nsP3:G3BP interactions

A MAYV nsP3 3D-model was built by homology using the NMR structure of the MAYV macrodomain (residues 1–159; PDB accession 5IQ5), and the C-domain was predicted *ab initio* using the Robetta server (Song et al., 2013). For all complex predictions, the crystal structure of the G3BP dimer (PDB accession 5FW5) was used after the removal of nsP3-SFV peptides. All docking simulations were performed using the ClusPro 2.0 server (Kozakov et al., 2017) using Piper as the fast Fourier transform (FTT)-based rigid docking program allowing a large positional sampling of nsP3. This step provides 10^9^ positions of nsP3 (70,000 rotations translated in x, y, z directions relative to G3BP) from which a thousand of representative rotation/translation combinations with low interaction energy were selected from ranking based on the scoring function (linear combinations of several energy terms that include attractive and repulsive van der Waals contributions describing shape complementarity and one more term representing the electrostatic part of the binding energy). ClusPro is attempting to find the most populated lowest energy solutions after clustering these solutions and ranking by cluster size. Analysis of interactions between G3BP and nsP3 of the best-ranked complex population (cluster with the largest number of members and exhibiting the lowest interaction energy) was carried out with the PyMOL Molecular Graphics System (v1.8, Schrödinger, LLC).

### Statistical analysis

All of the analyses (unpaired Student’s t-test) were performed using GraphPad Prism version 6 (GraphPad Software Inc.). *: p<0.05; **: p<0.01; ***: p<0.001.

## Data availability statement

The original contributions presented in the study are included in the article/supplementary material. Further inquiries can be directed to the corresponding authors.

## Author contributions

AN, EB, OA-R-P, WB, PE, and LC performed the experiments. LB supervised the overall project. LC and LB wrote the first draft of the manuscript. All authors contributed to the article and approved the submitted version.

## Acknowledgments

We thank Camille Clop for technical assistance in transfection experiments, and the Montpellier Resource Imaging platform (http://www.mri.cnrs.fr/en/) for confocal imaging facility. We are grateful to James Wegger-Lucarelli (Department of Biomedical Sciences and Pathobiology, Virginia Tech, MD USA) for providing us with the MAYV-luc reporter virus and to Andres Merits (Institute of Technology, University of Tartu, Estonia) for sharing the CHIKV-nanoLuc virus. We are indebted to Lise Chauveau for helpful discussions and critical reading of the manuscript. O.A.P. is a fellow of the French Ministry of Education, Research, and Innovation. W.B. was granted by Infectiopole Sud Foundation. The funders had no role in study design, data collection, and interpretation, or the decision to submit the work for publication.

## Conflict of interests

The authors declare that the research was conducted in the absence of any commercial or financial relationships that could be construed as a potential conflict of interest.

## Publisher’s note

All claims expressed in this article are solely those of the authors and do not necessarily represent those of their affiliated organizations, or those of the publisher, the editors and the reviewers. Any product that may be evaluated in this article, or claim that may be made by its manufacturer, is not guaranteed or endorsed by the publisher.
